# Rfam 14: expanded coverage of metagenomic, viral and microRNA families

**DOI:** 10.1093/nar/gkaa1047

**Published:** 2020-11-19

**Authors:** Ioanna Kalvari, Eric P Nawrocki, Nancy Ontiveros-Palacios, Joanna Argasinska, Kevin Lamkiewicz, Manja Marz, Sam Griffiths-Jones, Claire Toffano-Nioche, Daniel Gautheret, Zasha Weinberg, Elena Rivas, Sean R Eddy, Robert D Finn, Alex Bateman, Anton I Petrov

**Affiliations:** European Molecular Biology Laboratory, European Bioinformatics Institute, Wellcome Genome Campus, Hinxton, Cambridge CB10 1SD, UK; National Center for Biotechnology Information, National Library of Medicine, National Institutes of Health, Bethesda, MD 20894, USA; European Molecular Biology Laboratory, European Bioinformatics Institute, Wellcome Genome Campus, Hinxton, Cambridge CB10 1SD, UK; European Molecular Biology Laboratory, European Bioinformatics Institute, Wellcome Genome Campus, Hinxton, Cambridge CB10 1SD, UK; RNA Bioinformatics and High-Throughput Analysis, Friedrich Schiller University Jena, Leutragraben 1, 07743 Jena, Germany; European Virus Bioinformatics Center, Leutragraben 1, 07743 Jena, Germany; RNA Bioinformatics and High-Throughput Analysis, Friedrich Schiller University Jena, Leutragraben 1, 07743 Jena, Germany; European Virus Bioinformatics Center, Leutragraben 1, 07743 Jena, Germany; Faculty of Biology, Medicine and Health, University of Manchester, Oxford Road, Manchester, M13 9PT, UK; Université Paris-Saclay, CEA, CNRS, Institute for Integrative Biology of the Cell (I2BC), 91198, Gif-sur-Yvette, France; Université Paris-Saclay, CEA, CNRS, Institute for Integrative Biology of the Cell (I2BC), 91198, Gif-sur-Yvette, France; Bioinformatics Group, Department of Computer Science and Interdisciplinary Centre for Bioinformatics, Leipzig University, 04107 Leipzig, Germany; Department of Molecular and Cellular Biology, Harvard University, Cambridge, MA 02138, USA; Department of Molecular and Cellular Biology, Harvard University, Cambridge, MA 02138, USA; Howard Hughes Medical Institute, Harvard University, Cambridge, MA 02138, USA; John A. Paulson School of Engineering and Applied Science, Harvard University, Cambridge, MA 02138, USA; European Molecular Biology Laboratory, European Bioinformatics Institute, Wellcome Genome Campus, Hinxton, Cambridge CB10 1SD, UK; European Molecular Biology Laboratory, European Bioinformatics Institute, Wellcome Genome Campus, Hinxton, Cambridge CB10 1SD, UK; European Molecular Biology Laboratory, European Bioinformatics Institute, Wellcome Genome Campus, Hinxton, Cambridge CB10 1SD, UK

## Abstract

Rfam is a database of RNA families where each of the 3444 families is represented by a multiple sequence alignment of known RNA sequences and a covariance model that can be used to search for additional members of the family. Recent developments have involved expert collaborations to improve the quality and coverage of Rfam data, focusing on microRNAs, viral and bacterial RNAs. We have completed the first phase of synchronising microRNA families in Rfam and miRBase, creating 356 new Rfam families and updating 40. We established a procedure for comprehensive annotation of viral RNA families starting with *Flavivirus* and *Coronaviridae* RNAs. We have also increased the coverage of bacterial and metagenome-based RNA families from the ZWD database. These developments have enabled a significant growth of the database, with the addition of 759 new families in Rfam 14. To facilitate further community contribution to Rfam, expert users are now able to build and submit new families using the newly developed Rfam Cloud family curation system. New Rfam website features include a new sequence similarity search powered by RNAcentral, as well as search and visualisation of families with pseudoknots. Rfam is freely available at https://rfam.org.

## INTRODUCTION

Rfam is the database of non-coding RNA (ncRNA) families (1), each represented by a multiple sequence alignment (known as the seed), a consensus secondary structure, and a covariance model to annotate non-coding RNAs in nucleotide datasets using the Infernal software (2). Rfam and Infernal are commonly used to annotate ncRNAs in newly sequenced genomes (3,4), and are the core components of the genome annotation pipelines in Ensembl (5), Ensembl Genomes (6), NCBI Prokaryotic and Eukaryotic Gene Annotation (7,8) and other resources. For example, PDBe uses Rfam to enable searching for RNA chains, such as tRNA or rRNA, in 3D structures (9), while RNAcentral uses Rfam to detect incomplete sequences, RNA type annotations errors, and provide other quality controls (10). The manually curated multiple sequence alignments from Rfam are also used for training and benchmarking new software, such as secondary structure prediction algorithms (11,12).

Here we describe community-driven improvements and updates that are available in Rfam 14 (releases 14.0–14.3), including new RNA families, new features on the Rfam website, and new tools for expert users to contribute families to the database. Rfam 14.3 contains 3444 families, representing a 28% increase since version 13.0. The majority of the new families have been generated in collaboration with other RNA resources and experts, in particular ZWD, miRBase and European Virus Bioinformatics Center (EVBC), supplemented by in-house literature curation. Following the transition from annotating a subset of ENA (13) to annotation of a collection of non-redundant and complete genomes in Rfam 13.0 (1), we have expanded the set of genomes in the Rfam sequence database (Rfamseq) by 76% in Rfam 14.0 to reflect the increase in number of genomes. Rfamseq now includes 14 772 genomes from all domains of life. Here, we also describe the newly-developed Rfam Cloud curation pipeline, which marks a further major step towards ensuring that Rfam remains a core open and sustainable resource for the whole RNA community.

## NEW FAMILIES FROM ZWD

**Figure 1.**
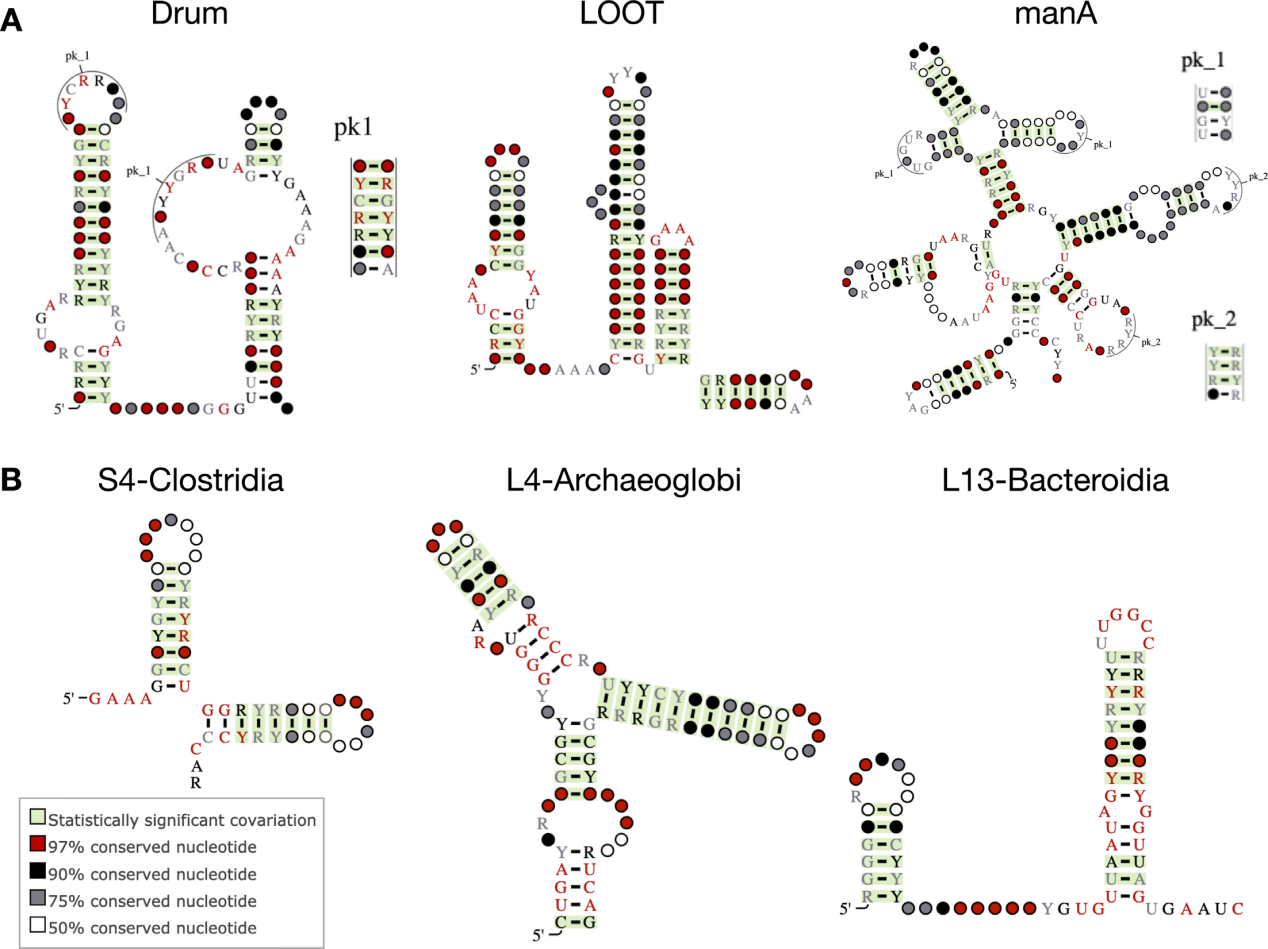
Example ZWD-based Rfam families. (**A**) Metagenomic-based RNAs Drum (RF02958), LOOT (RF03000), and manA (RF01745) RNAs; (**B**) S4-Clostridia (RF03140), L4-Archaeoglobi (RF03135) and L13-Bacteroidia (RF03127) r-leader families. All families have been created in releases 14.1 and 14.3, except for manA, which was updated in 14.3. Purines (adenine or guanine) are shown as ‘R’, while pyrimidines (cytosine or uracil) are shown as ‘Y’.

Rfam releases 14.1 and 14.3 included 253 new families from the Zasha Weinberg Database (ZWD) that were discovered by a systematic computational analysis of intergenic regions in Bacteria and metagenomic samples (14), as well as newly identified ribosomal leaders (r-leaders) (15). ZWD stores the original manually curated multiple sequence alignments produced in the Breaker and Weinberg groups over the last decade. ZWD is a git-based resource that currently includes 417 alignments and is available at https://bitbucket.org/zashaw/zashaweinbergdata. Examples of the new and updated ZWD-based families are shown in Figure 1.

Prior to version 14, Rfam had incorporated 108 families from ZWD, but in Rfam 14 a new process was developed to automate the addition of ZWD alignments. Since the inception of Rfam in 2003, it has been a strict requirement that sequences in seed alignments were derived from Rfamseq, the underlying sequence database that is searched by covariance models for all families with each release. The Rfamseq database sequences must also exist in public databases like GenBank or ENA and each seed sequence is automatically checked to ensure that it is a valid subsequence of a publicly available sequence. However, many of the ZWD families come from environmental samples, so the sequences were not found in the INSDC archives. Previously such sequences were replaced with closely related ones from INSDC or removed, which required modifying the user-submitted alignments and could result in smaller alignments missing covariation compared to the originals. In order to preserve the manually curated alignments as much as possible and avoid the error-prone manual steps, we imported all ZWD sequences into RNAcentral (10) to create stable accessions and allowed for the RNAcentral identifiers to appear in Rfam seed alignments. Furthermore, we have updated the Rfam pipeline to allow seed alignment sequences to derive from any valid ENA or GenBank accession. This added flexibility not only allowed us to import the ZWD families, but will facilitate the construction of seed alignments, especially using the new cloud-based Rfam family building pipeline (see below).

To confirm the completeness of the import, we systematically compared Rfam families with ZWD using the Infernal cmscan program (2) to search all ZWD sequences with Rfam covariance models. Ninety eight percent of ZWD alignments have now been imported into Rfam except for seven families lacking covariation support as determined by R-scape (16) and 43 families marked ‘Not for Rfam’ in ZWD. The mapping between ZWD alignments and Rfam accessions is provided in Supplementary Information.

We also used CaCoFold (17) to identify the pre-existing ZWD-based Rfam families requiring an update using the new RNAcentral import mechanism. For example, the manA alignment from Rfam (RF01745) had only 29 statistically significant basepairs while a CaCoFold structure based on the corresponding alignment in ZWD contained 48 significant basepairs. A comparison between the Rfam and ZWD versions of the alignment revealed a mistake in the Rfam secondary structure introduced during manual import. As a result, the manA Rfam alignment was updated to its original version from ZWD (Figure 1A, right). We are systematically reviewing other ZWD-based families that may require an update and will release them in future Rfam versions (17).

## NEW WORKFLOW FOR VIRAL RNA FAMILIES

Despite the small size (3400–41 000 nucleotides) of RNA virus genomes, they contain several conserved RNA structures vital for protection against exonucleases, genome diversification, and play a crucial role in various stages of their viral life cycle (18). Many viruses rely on RNAs to infect and replicate inside a host: for example, the *cis*-acting element of coronaviruses is essential for replication (18), while in Dengue viruses replication depends on RNA structure-mediated circularization (19,20). RNA viruses are often highly contagious and can lead to fast-emerging severe diseases (21). Being able to identify and understand viral RNAs is essential for the scientific community to develop novel drugs and treatments in response to pandemics like COVID-19.

In releases 14.2 and 14.3, Rfam created 22 new families, focusing on *Coronaviridae* and *Flavivirus* structured RNAs. These releases are the first of many planned extensions for viral RNA families in Rfam. We will continually update the functional RNA structures of viral clades in Rfam and aim to provide a comprehensive database for virologists interested in RNA secondary structures. This effort is the first of its kind to bring bioinformaticians and virologists together to publish detailed and specific RNA alignments and secondary structures for a broad range of RNA viruses, and thus complements protein-based databases like vFam (22) and RVDB (23).

### *Coronaviridae* families

**Figure 2.**
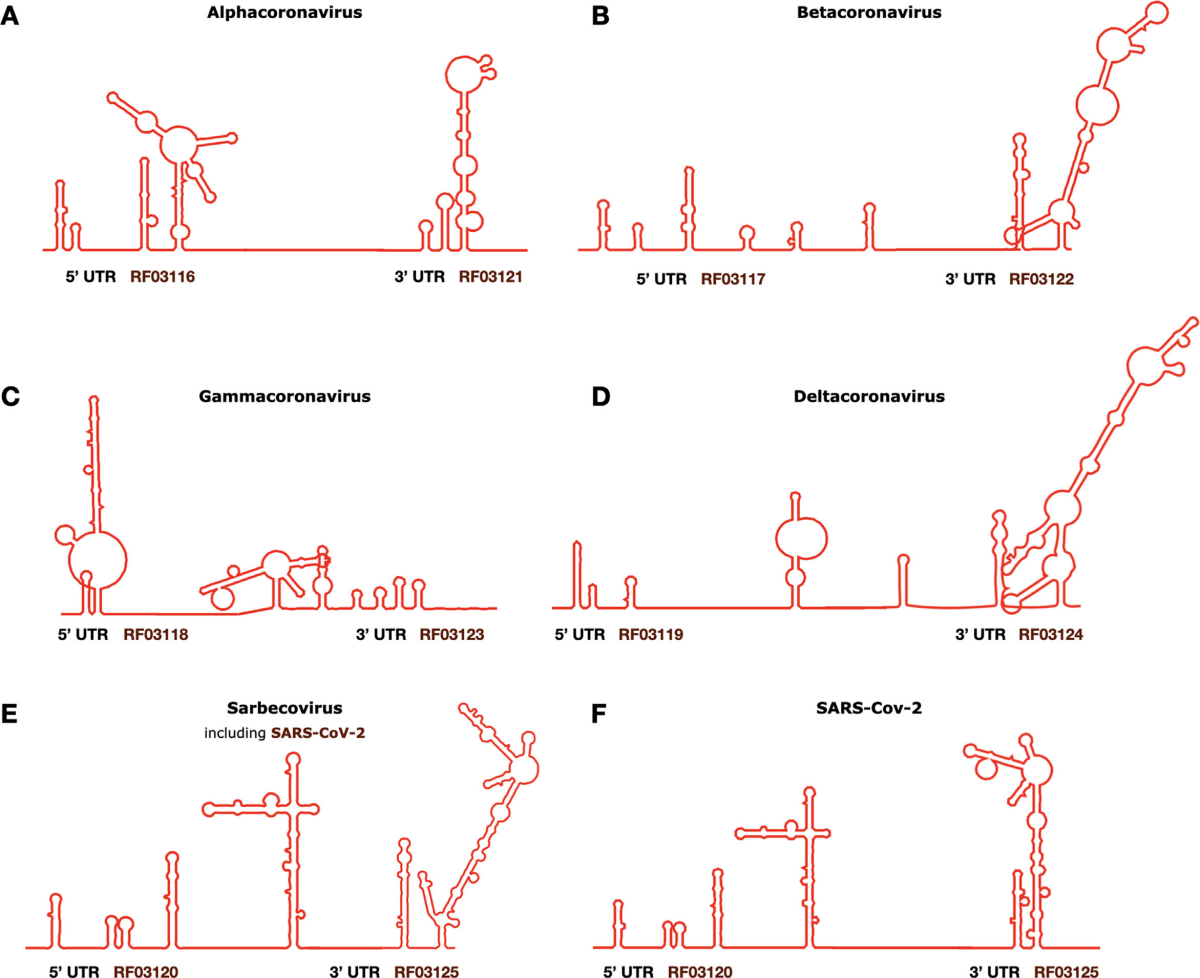
New Rfam *Coronaviridae* 5′ and 3′ UTR families are depicted schematically within the complete viral genomes. (**A**) *Alphacoronavirus* UTRs (RF03116 and RF03121); (**B**) *Betacoronavirus* UTRs (RF03117 and RF03122); (**C**) *Gammacoronavirus* UTRs (RF03118 and RF03123); (**D**) *Deltacoronavirus* UTRs (RF03119 and RF03124); (**E**) *Sarbecovirus* UTRs (RF03120 and RF03125); (**F**) visualising SARS-CoV-2 UTRs using the *Sarbecovirus* Rfam models.

In response to the SARS-CoV-2 outbreak, the Rfam team prepared a special release 14.2 dedicated to the *Coronaviridae* RNA families (24), including ten new and four revised families that can be used to annotate SARS-CoV-2 and other *Coronaviridae* genomes with RNA families. The new RNA families represent the entire 5′- and 3′- untranslated regions (UTR) for *Alpha-*, *Beta-*, *Gamma-* and *Deltacoronavirus* subfamilies (Figure 2A–D). The families were built based on a set of high-quality alignments produced with LocARNA (25) and reviewed by expert virologists from the Marz group (University of Jena) and the EVBC. In addition, a set of alignments was built for the *Sarbecovirus* subgenus UTRs, including the SARS-CoV-1 and SARS-CoV-2 UTRs (Figure 2E). While the *Alpha*-, *Beta*- and *Deltacoronavirus* alignments and structures were refined based on the literature (26–28), the *Gammacoronavirus* families are based on prediction alone due to the lack of experimental data.

We also reviewed and updated the existing *Coronaviridae* Rfam families, including Coronavirus packaging signal (RF00182), Coronavirus frameshifting stimulation element (RF00507), Coronavirus s2m RNA (RF00164), and Coronavirus 3′-UTR pseudoknot (RF00165). Two Rfam families were superseded by the new whole-UTR alignments and removed from the database: the Coronavirus SL-III cis-acting replication element (RF00496) representing a single stem that is now found in *Alpha*- and *Betacoronavirus* 5′-UTR families (aCoV-5UTR and bCoV-5UTR) and Coronavirus_5p_sl_1_2 (RF02910) representing two stems from aCoV-5UTR. The new and updated *Coronaviridae* Rfam families are available at https://rfam.org/covid-19.

### *Flavivirus* families

In order to create new *Flavivirus* families, we identified a set of full-length, non-redundant *Flavivirus* genomes to serve as a source of sequences. All *Flavivirus* genomes marked as ‘complete’ were downloaded from the ViPR database (10 443 genomes as of August 3rd, 2020) (29). Since most of the known flaviviral genomes are represented by the Dengue virus genomes (∼58%), the ViPR-based Dengue sequences were excluded and the curated high-quality RefSeq sequences were used instead. The genomes were scanned with the existing Rfam models for Dengue virus SLA (RF02340), *Flavivirus* capsid hairpin cHP (RF00617), and *Flavivirus* 3′ UTR CRE (RF00185) to identify the genomes with complete 5′ and 3′ UTRs. The resulting 2661 genomes were used to refine the existing *Flavivirus* Rfam families as well as previously published models (30) to produce a set of 12 new and 2 updated Rfam families (Table 1).

**Table 1.** New and updated *Flavivirus* RNA families from release 14.3. The two updated families are marked with an asterisk

Rfam accession	Rfam ID	Rfam description
RF03546	Flavivirus-5UTR	Flavivirus 5′ UTR
RF00185*	Flavi_CRE	Flavivirus 3′ UTR cis-acting replication element (CRE)
RF00525*	Flavivirus_DB	Flavivirus DB element
RF03547	Flavi_xrRNA	General Flavivirus exoribonuclease-resistant RNA element
RF03545	Flavi_ISFV_CRE	Insect-specific Flavivirus 3′ UTR cis-acting replication element (CRE)
RF03544	Flavi_ISFV_repeat_Ra	Insect-Specific Flavivirus 3′ UTR repeats Ra
RF03543	Flavi_ISFV_repeat_Rb	Insect-Specific Flavivirus 3′ UTR repeats Rb
RF03542	Flavi_ISFV_repeat_Ra_Rb	Insect-Specific Flavivirus 3′ UTR repeats Ra and Rb elements
RF03541	Flavi_ISFV_xrRNA	Insect-specific Flavivirus exoribonuclease-resistant RNA element
RF03540	Flavi_NKV_CRE	No-Known-Vector Flavivirus 3′ UTR cis-acting replication element (CRE)
RF03539	Flavi_NKV_xrRNA	No-known vector Flavivirus exoribonuclease-resistant RNA element
RF03538	Flavi_TBFV_CRE	Tick-borne Flavivirus 3′ UTR cis-acting replication element (CRE)
RF03537	Flavi_TBFV_SL6	Tick-borne Flavivirus short stem-loop SL6
RF03536	Flavi_TBFV_xrRNA	Tick-borne Flavivirus exoribonuclease-resistant RNA element

A single Rfam family was generated for the complete *Flavivirus* 5′ UTR (RF03546) to respect the conserved order of the structural elements SLA and SLB. However, a single model could not represent the variability of the 3′ UTR as it can vary both in terms of structural composition and length (from 400 to 900 nucleotides). Therefore we produced three general 3′ UTR models that are valid for all viruses of the *Flavivirus* genus and represent the xrRNA, CRE, and DB elements (RF03547, RF00185, and RF00525), as well as a set of specialised models for the viral clades based on their host-range (31,32). For insect-specific flaviviruses (**ISFV**), we created two specialised models: CRE and xrRNA (RF03545 and RF03541) with increased sensitivity for ISFVs, as well as three families unique to ISFV: the repeated elements (Ra and Rb) individually (RF03544 and RF03543) and as a combined element (RF03542) (30,33). In most instances, Ra and Rb co-occur. However, in some ISFVs (e.g. Quang Binh virus (QBV) and Mosquito flavivirus (MSFV)), the Rb element is missing from some repeats (30). Furthermore, there may be novel as yet undescribed *Flavivirus* species with another repeat pattern. Therefore, the individual repeated elements and the co-occurring model were created and integrated into the Rfam.

We provide three tick-borne flavivirus (**TBFV**) models, enabling more sensitive searches for the CRE (RF03538) and xrRNA (RF03536) elements, as well as the SL6 stem (RF03537) that appears in some 3′ UTRs in TBFVs (30,34). Although there is no known vector (**NKV**) for some flaviviruses, they can still be grouped and annotated using the specialised CRE and xrRNA models (RF03540 and RF03539). The remaining host-specific group, the mosquito-borne flaviviruses (**MBFV**), will be imported in a future Rfam release, once the detailed analysis becomes available in the literature. Finally, two *Flavivirus* families (RF00465 and RF02549) have been removed from Rfam since they were superseded by the new families.

### Generalising the workflow for annotating viral RNAs in Rfam

We are generalising the procedure established for the *Coronaviridae* and *Flavivirus* families to annotate structured RNAs in all viruses, starting with the human pathogens like *Hepacivirus* (Hepatitis C viruses), *Filoviridae* (e.g. *Ebolavirus*) and *Rhabdoviridae* (e.g. Rabies viruses). The method is based on clustering the set of available complete genomes in ViPR (29) and RefSeq (8) and calculating high-quality genome alignments of representative viruses. These alignments are refined by local RNA secondary structure information using LocARNA (35). The structurally conserved elements, as identified by RNAz (36), are extracted from the alignment, compared with the literature and manually curated by expert virologists from the EVBC before submission to Rfam. The new viral families will enable researchers to rapidly annotate viral genomes with conserved RNA structures using the Infernal software and Rfam covariance models, detect structured non-coding viral regions in metagenomic data, and gain insight into the recombination of the conserved RNA structures (37).

## SYNCHRONISING MICRORNA FAMILIES BETWEEN RFAM AND MIRBASE

MicroRNAs are a class of ∼22 nt ncRNA that regulate gene expression at the post-transcriptional level. Animal and plant genomes contain hundreds to thousands of microRNA genes, many of which have been implicated in processes such as development and disease (38). For example, the mir-17-92 cluster and mir-155 have been shown to act as oncogenes [oncomirs; reviewed in (39)]. Understanding the evolutionary relationships between microRNAs of different species allows the transfer of gene annotation and functional information, for example from model organisms to human. MicroRNA sequences and annotations are aggregated in miRBase (40), the authoritative resource for published microRNA genes.

miRBase is primarily a sequence database, but both Rfam and miRBase contain classifications of microRNA families. However, before Rfam 14.3 the two databases have not been coordinated or synchronised. Previously, miRBase used a semi-automated, clustering method relying on BLAST (41). These sequence-only miRBase families have higher coverage but lower quality than the Rfam microRNA families. In release 14.2, Rfam contained 529 microRNA families, while miRBase v22 annotated 1,983 microRNA families. Only 28% of the miRBase families matched one or more of the Rfam 14.2 families. There was therefore an opportunity to create up to 1500 new families to increase the coverage of microRNAs in Rfam, as well as investigate and rationalise the entries that are unique to each database. Here, we present the first phase of a comprehensive review and classification of microRNA gene families in collaboration with miRBase.

Based on miRBase v22, we have manually curated an initial set of 1678 multiple sequence alignments. The remaining ∼300 families require more detailed consideration and curation, including merging and splitting, and will be revisited in a second phase. The manually curated alignments were used as seeds for building the covariance models, with which we searched the Rfam sequence database for homologs of these new microRNA families. At the time of writing, we have created and submitted 356 new microRNA families to Rfam and updated 40 existing families. Work is underway to create and review the remaining microRNA families, which will be made available in the subsequent Rfam release.

The workflow established here will rationalise microRNA families in the key RNA database resources, and ensure consistency between Rfam, miRBase, and RNAcentral. miRBase will retain its focus on sequences, and Rfam will be the primary resource of microRNA family classifications. These Rfam microRNA family classifications will be made available in both miRBase and RNAcentral. The relationship between the databases will create a cycle of improvement. All Rfam microRNA seed alignments will contain only sequences that are validated as microRNAs by miRBase. Rfam microRNA families will be used to identify new member sequences, and these new sequences will be reviewed by miRBase for inclusion in both miRBase and RNAcentral. The validated microRNA sequences will then be added to an updated version of the Rfam seed alignment. Since miRBase and Rfam are both RNAcentral member databases, the synchronisation of miRBase and Rfam is facilitated by consistent use of RNAcentral sequence identifiers for all sequences in microRNA seed alignments. The new Rfam models coupled with Infernal will enable other resources, including the key genome browsers and model organism databases, to annotate microRNA sequences in genome sequences in a rigorous and sustainable way.

## CLOUD-BASED FAMILY CURATION SYSTEM

Creating new Rfam families is a computationally expensive process that depends on searching the Rfam non-redundant collection of complete genomes (360Gb as of release 14.3) using Infernal (2). Such searches are impractical without access to storage, memory, and CPU resources that can parallelise the execution and reduce the running time. To enable the scientific community to build Rfam families without setting up their own computational infrastructure, the Rfam curation pipeline was packaged in software containers using Docker and deployed using Kubernetes, a container orchestration engine that automates cloud deployment and manages containerised applications. The new cloud-based family curation pipeline, Rfam Cloud, is hosted at the Embassy Cloud platform provided by EMBL-EBI.

Rfam Cloud provides access to a command line environment that enables users to create or modify Rfam families. The Rfam family building process involves several steps: starting with a single sequence or a seed alignment containing known examples of a family, the user can search for similar sequences using a covariance model, refine the family by adding more sequences into the seed alignment, and identify a bit score cutoff (the gathering threshold) that separates homologous sequences that constitute an Rfam family from non-homologous sequences. This process can be iteratively repeated to improve the seed alignment and the associated covariance model. The user can also perform quality control checks to verify the format and ensure that there are no overlaps with existing families. Upon successful completion of quality control steps, new families can be submitted to the Rfam team for review and inclusion in the main Rfam database. The Rfam Cloud documentation describes the curation tools and provides guidelines and tips for common tasks, such as selecting the gathering threshold for a family and is available at https://rfam.org/cloud.

Rfam Cloud enables RNA family building using Rfam's Infernal-based search pipeline and requires some manual intervention between iterations. Two alternative RNA family building tools with more automation than the Rfam Cloud pipeline are GraphClust2 (42) and RNAlien (43), both of which also use Infernal amongst other tools. However, Rfam Cloud is directly linked to the Rfam database where the nascent families can be deposited and accessed by the larger community.

Following a testing period in 2019, Rfam Cloud was successfully used in a higher education setting in collaboration with the University of Paris-Saclay. A three-month masters level RNA bioinformatics course was held from October 2019 to January 2020, where eight teams of three graduate students used Rfam Cloud to build RNA families. Each team was assigned one candidate sequence to initiate the family building process. The students used Rfam Cloud at their own pace and were provided support using a Slack workspace where the Rfam team answered questions and helped with troubleshooting. The students produced 6 new Rfam families (RF03530-RF03535) that are listed in Table 2.

**Table 2.** New Rfam families created during an Rfam course at University of Paris-Saclay

Rfam accession	Rfam ID	Rfam description	Reference
RF03530	bglG-cis-reg	*cis*-regulatory element of the bglG/LicT operon	(44)
RF03531	n00280_RNA	*Clostridioides difficile* sRNA included into helicase gene	(44)
RF03532	SQ2397_RNA	*cis*-regulator of HTH transcription factor	(44)
RF03533	SQ1002_RNA	*Clostridioides difficile* sRNA SQ1002	(44)
RF03534	sRNA71	*Staphylococcus* sRNA71 small RNA	(45)
RF03535	TEG147	*Staphylococcus aureus* small RNA TEG147	(45)

The Rfam Cloud pipeline is currently used by a group of 15 external users contributing to family curation. Following approved requests, users are provisioned with a private cloud account where they can access the Rfam curation tools through a command line interface. New accounts can be requested at https://rfam.org/cloud.

### Getting credit for authoring Rfam families using ORCID

The ORCID registry (https://orcid.org) provides unique identifiers that unambiguously link researchers with their scientific papers and other outputs. The Rfam database is now integrated with the ORCID system, allowing authors of Rfam families to get credit for their contributions by adding Rfam family accessions to their ORCID profiles. The ORCID identifiers have been manually associated with existing Rfam families and the new families. This feature was enabled by the ‘Claim to ORCID’ functionality provided by the EBI Search (46). The process includes three steps: (a) search for an ORCID identifier on the EMBL-EBI website; (b) manually select all or a subset of listed entries and click ‘Claim to ORCID’; (c) login to ORCID using the same ORCID identifier and agree to add the Rfam entries to the ORCID record. As the RNA community begins using the Rfam Cloud infrastructure, this integration will allow the growing number of Rfam contributors to get credit for their work.

## OTHER IMPROVEMENTS

### Pseudoknot search and visualisation

**Figure 3.**
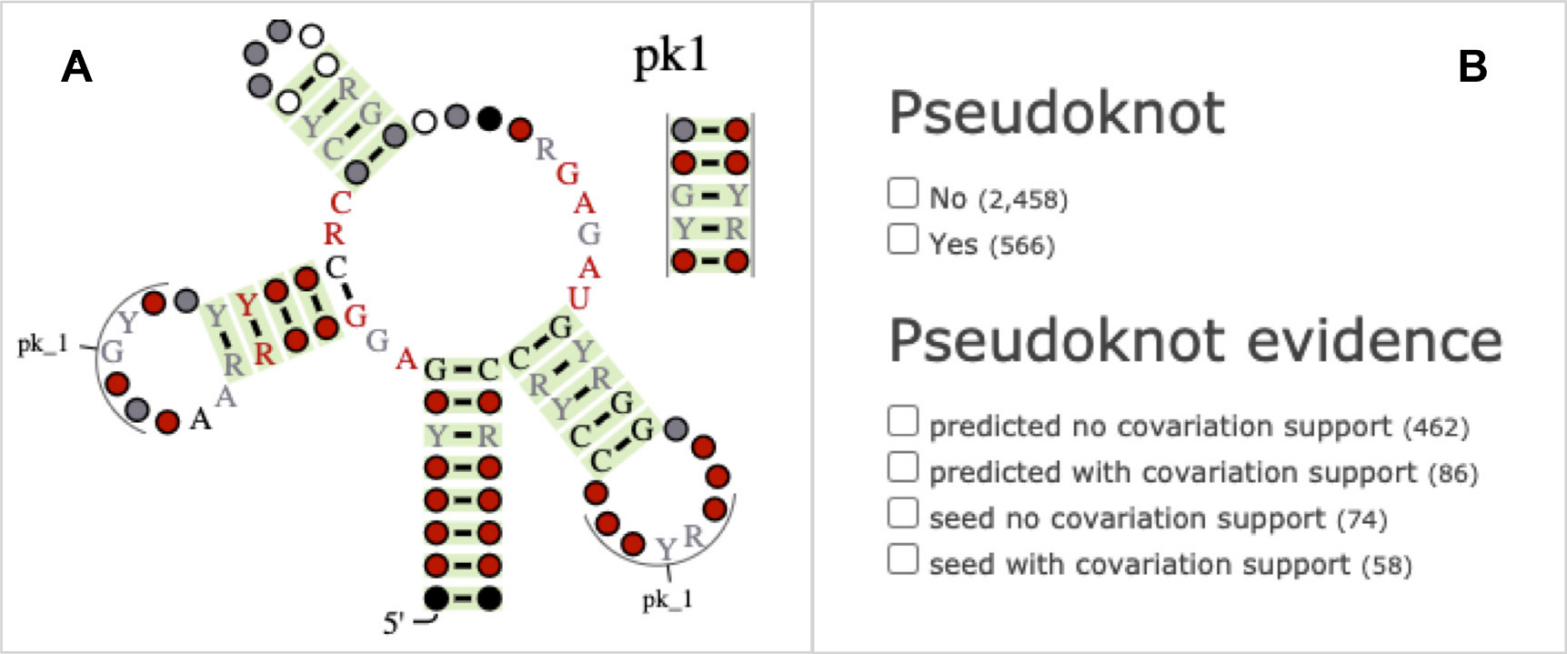
(**A**) R-scape visualisation of the skipping-rope RNA (RF02924). The nucleotides forming the pseudoknot are labelled *pk_1* and are shown as a separate stem. The basepairs with significant covariation, according to R-scape, are colored green. (**B**) Pseudoknot facets from the Rfam text search.

The R-scape software analyses covariation support for RNA secondary structure based on multiple sequence alignments (16). Starting with version 1.2.0, R-scape systematically identifies pseudoknots and other non-nested interactions provided that they have covariation support (17) and displays them using R2R (47). We analysed all Rfam seed alignments with R-scape and added pseudoknot visualisation to the Rfam website (Figure 3A). In addition, we updated the Rfam text search to enable searching for families with or without pseudoknots and filter the results by whether the pseudoknots have covariation support (Figure 3B).

### New sequence search integrated with RNAcentral

**Figure 4.**
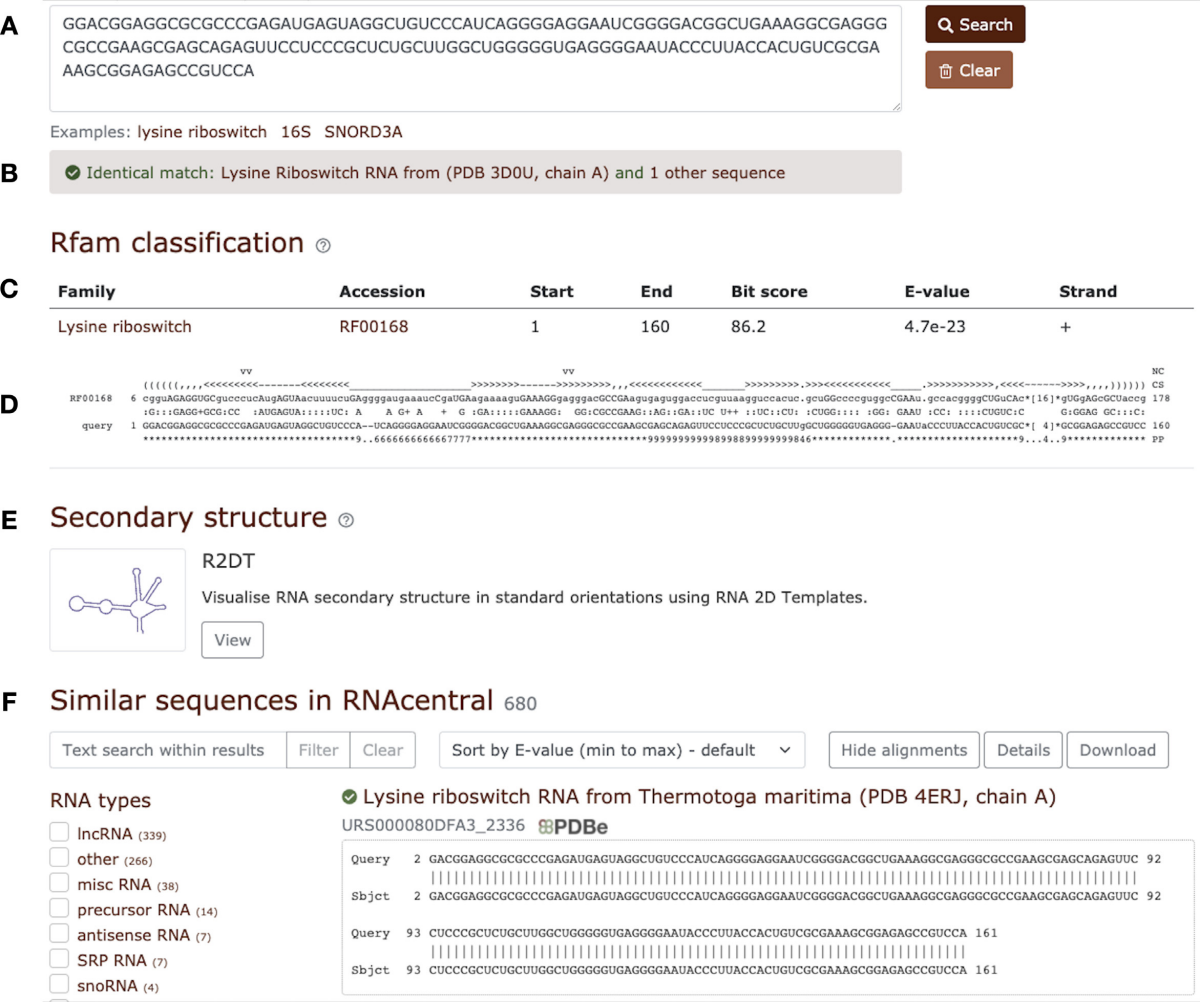
Rfam sequence search using the RNAcentral sequence search component. (**A**) Query sequence. (**B**) A sequence identical to the query found in RNAcentral. (**C**) Rfam classification using Infernal. (**D**) Alignment between the query and the Rfam covariance model. (**E**) Secondary structure visualised using R2DT displayed using the consensus secondary structure from the corresponding Rfam model. Clicking the link opens a new window with the detailed secondary structure diagram. (**F**) Similar sequences from RNAcentral.

The Rfam sequence search has been updated to use the RNAcentral reusable sequence search component. The search is executed on the RNAcentral cloud infrastructure and, in addition to annotating the query sequence with Rfam families using Infernal, identifies similar sequences in the RNAcentral sequence database using nhmmer (48). The new search also performs the clan competition procedure (49) which selects the longest and highest scoring hit if several Rfam families from the same clan match the query sequence. The new search can show related RNAs even if a query sequence does not match any Rfam families (e.g. most lncRNA queries will not have matches in Rfam). The search is also integrated with R2DT (50) to visualise RNA secondary structure in standard, reproducible, and recognisable layouts (Figure 4E).

The user interface features facets that enable filtering similar sequences by RNA type, organism, or the source database (Figure 4F). The results can also be filtered with any keyword and exported for further processing. The search is available under the sequence search tab at https://rfam.org/search.

## CONCLUSIONS

After eighteen years of development, Rfam still continues to grow as new RNA families are regularly reported in the literature. The recent innovations and improvements described here are focused on collaboration with the RNA community and other RNA resources to share data and tools to classify RNA families. Furthermore, the new Rfam Cloud pipeline is designed to involve more RNA experts in the creation of new families and narrow the gap between cutting edge research and manual database curation. Our future plans, in addition to completing the microRNA and viral projects, include the integration of experimentally determined 3D structure information into seed alignments and connecting the existing RNA families with the latest literature using text mining. The new Rfam Cloud and ongoing collaborations with resources such as ZWD, EVBC and miRBase cements Rfam's position as the community resource for RNA families. We invite new data submissions and feedback at https://docs.rfam.org/page/contact-us.html.

## DATA AVAILABILITY

Rfam is available under the Creative Commons Zero (CC0) license at https://rfam.org. The data can be accessed in the FTP archive, as well as through an API and a public MySQL database (see https://docs.rfam.org and (51) for instructions). All code is available on GitHub under the Apache 2.0 license at https://github.com/Rfam.

## Supplementary Material

gkaa1047_Supplemental_File

## Notes

Present address: Joanna Argasinska, Stanford University; Department of Genetics; 3165 Porter Drive, Palo Alto, CA 94304, USA.
